# Supplementary material for: Full thermoelectric characterization of a single molecule
Source: Nat Commun. 2023 Jun 30;14:3868. doi: 10.1038/s41467-023-39368-7 (PMC10313753; doi:10.1038/s41467-023-39368-7)
Supplement: Supplementary file 1 — Supplementary Information [file 41467_2023_39368_MOESM1_ESM.pdf]

Supplementary Information
Full thermoelectric characterization of a single molecule.

Andrea Gemma[1], Fatemeh Tabatabaei[2], Ute Drechsler[1], Anel Zulji[1], Herve Dekkiche[3], Nico Mosso[1], Thomas Niehaus[2], Martin R. Bryce[3], Samy Merabia[2] and Bernd Gotsmann[1]*

[1] IBM Research Europe– Zurich, 8803 Rueschlikon, Switzerland
[2] Université Claude Bernard Lyon 1, CNRS, Institut Lumière Matière, F-69622 Villeurbanne, France
[3] Department of Chemistry, Durham University, Durham, DH1 3LE, United Kingdom

*Corresponding author: bgo@zurich.ibm.com

The molecule

The molecule under test is a derivative of the well-known oligo(phenyleneethynylene) (OPE3) compound (3 denotes the number of phenylene rings in the backbone) with an anthracenyl group (-An) as the central ring. At each extreme, the molecule features a dihydrobenzo[*b*]thiophene (DHBT) group as anchor point for the metal electrodes. This group is known to bind efficiently to gold electrodes, resulting in a high junction formation probability[1,2,3], without the problems associated with the more common thiol group, such as oxidation and formation of metal-thiolate clusters [4]. The reason for selecting the OPE3-An molecule is due to its high thermal stability, as already observed in a previous thermoelectric study by Dekkiche *et al.* [5], and in general to the properties of OPE3 derivatives to be synthetically-versatile, highly-conjugated, robust molecules with a large consensus in the community about their electrical properties[6].

Meaning of *zT* and Efficiency

By combining the three measured quantity as described in eq.1 in the main text it is possible to determine experimentally the figure-of-merit *zT.*

The figure of merit indicates how close a system is to the theoretical maximum efficiency, i.e. a Carnot machine working between the same temperature difference. The optimum condition for maximum efficiency is indeed:

$$\eta_{max} = \frac{T_H - T_C}{T_H} \cdot \frac{\sqrt{1+zT}-1}{\sqrt{1-zT}+T_C/T_H}$$

where T_H and T_C are the hot- and cold-side temperatures, respectively. The thermoelectric conversion efficiency is the product of the Carnot efficiency ($T_H - T_C/T_H$) and a reduction factor as a function of the material's figure of merit zT, taken at $T = T_{amb} + \frac{\Delta T}{2}$, where $\Delta T = T_H - T_C$

κ as difference of fits and thermal background slope

A plateau of constant thermal conductance is usually not observed in the thermal signal [7,8]. Instead, a linear decrease versus tip displacement is usually present, with slopes ranging between 0.5 and 2 nW/(K nm). This is caused by spurious thermal conductance paths around the actual molecular junction, that forms a thermal background, which is modulated by tip retraction. As already pointed out by Mosso et al. [7], those paths can be attributed essentially to three effects. The first effect is the thermal near-field radiation occurring between the tip and the platform even for nominally clean surfaces [9,10] . The second is due to the presence of possible solvent residues which, despite the cleaning procedure, can still be present on the surface. The third contribution comes from the deposited molecules themselves, that are not directly bound to the electrodes but can anyway establish a thermal contact through Van der Waals bonds [11,12,13]. A plateau of constant thermal

conductance is usually not observed in the thermal signal. Indeed, a linear decrease versus tip displacement is usually present, with slopes ranging between 0.5 and 2 nW/(K nm). This is caused by spurious thermal conductance paths around the actual molecular junction, that forms a thermal background, which is modulated by tip retraction. As already discussed in details in Mosso *et al.* [7], those paths can be attributed to three effects. First, even nominally clean gold surfaces can show significant heat transfer of this magnitude due to thermal near-field radiation [9,10] . The magnitude of this contribution is currently under debate [9,10]. It is worth to mention that, recently, thermal conductance variations vs. tip−surface distance have been reported in near-field measurements in UHV, even after thorough in situ cleaning procedures. Those measurements exhibit background slopes between 0.5 and 8 nW/K/nm, close to the range reported in this study [9,10]. Those measurements suggest that contributions due to radiative processes at nm distances might not be ignored, and most likely might not be avoided experimentally. Second, despite the cleaning processes, there still might be residues and/or contaminants on the surface coming from the MEMS fabrication steps and exposure to the solvent. Third, also the deposited molecules that are not directly bound to the electrodes can establish a thermal contact. All those adsorbates appear electrically inactive, because only weakly bonded to the electrodes, but able to carry significant heat, on the order of 10−30 nW/K, via Van der Waals bonds [11,12,13]. Due to the differential scheme for measuring the thermal conductance, the reproducibility and the slope of the thermal background signal are more important than its magnitude. Within our experimental conditions, the thermal background results stable and does not vary significantly during repeated cycles of the break-junction event, remaining approximately linear in the region of few ångströms around the breaking point of the molecular plateau. On a larger length scale, however, there is no prior assumption on the linearity of the background signal.

Phononic heat flux spectrum

To further analyze the vibrational modes involved in phonon thermal transport across the molecule, we have also computed the phonon spectral thermal conductance using MD simulations [14,15]. Figure 4 compares the thermal conductance of DHBT-OPE3-An and DHBT-OPE3 molecule junctions. Note that due to the low Debye frequency of gold (~260 cm^{-1}) [16]phonons with energies higher than 260 cm^{-1} are filtered out and not included in the histogram. Below that frequency, the two spectra match relatively well except at low frequencies (Figure S1). The relative discrepancies observed are related to the effect of the pending groups which should act here as a low frequency filter.

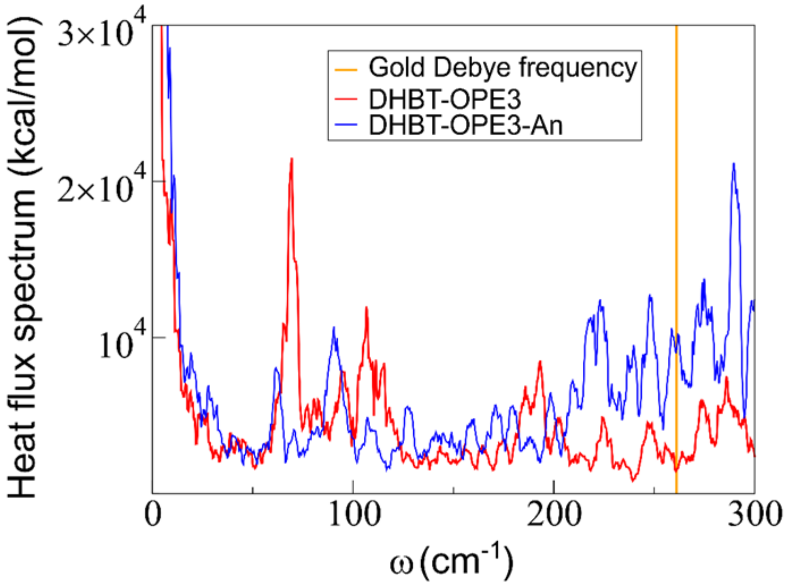

Figure S1. Comparison of the phonon heat flux spectrum of DHBT-OPE3-An (blue curve) and DHBT-OPE3 (red curve) molecule junctions obtained with molecular dynamics simulations. The orange line at 260 cm^{-1} is the Debye frequency of the two gold electrodes.

Rigidity of the molecule

To confirm the rigidity of the backbone of the OPE3 derivative, the torsion of the molecule junctions has been studied by means of MD simulations. The torsion angles between phenylene groups in the course of a 20 ns trajectory did not exceed 10 degrees and the DHBT-OPE-An remained planar [17]. Our studies show that the metal-molecule interface has a high conformational rigidity due to the DHBT anchor group [14].

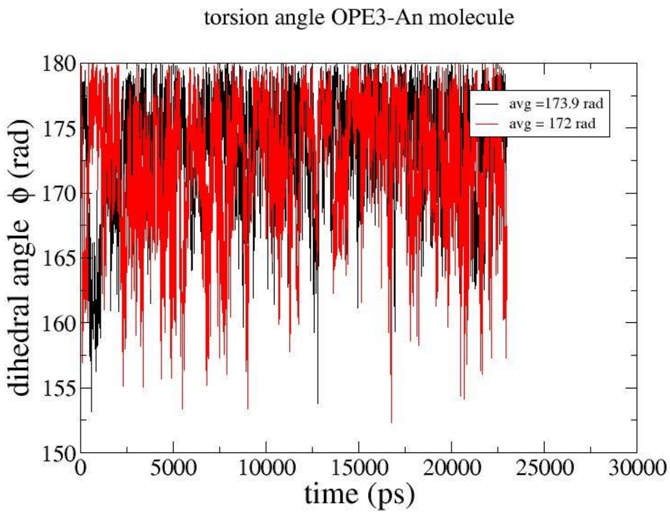

Figure S2. Torsion angle for DHBT-OPE3-An molecule.

Comparison with literature

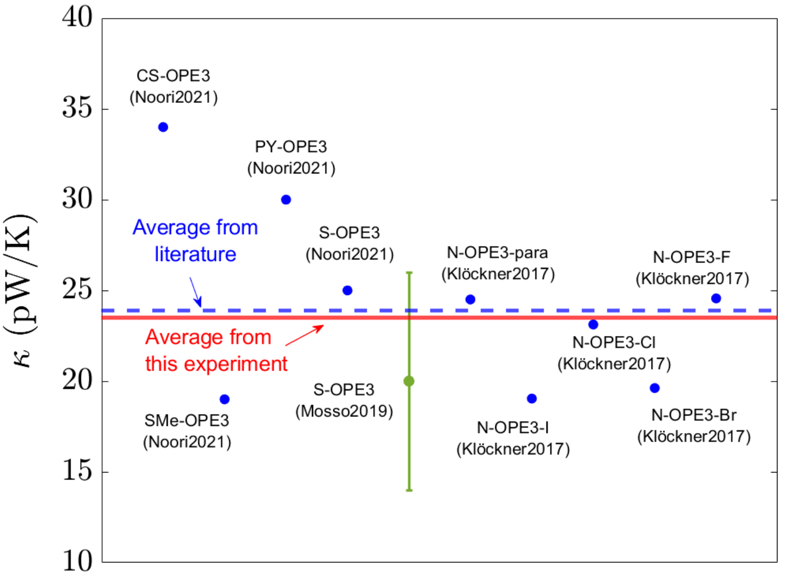

Figure S3. Comparison of κ values for DHBT-OPE3-An molecule with other OPE3 based molecules from the literature. Blue dots represent theoretical prediction, while the green dot is the experimental value. The average of the two samples analyzed in this study is represented by the red line.

Reference measurement using SAc-OPE3

In order to verify the protocol to measure the Seebeck coefficient, it was also applied to the same molecule that was previously examined in the same setup, namely the OPE3 molecule with standard thioacetate end groups (SAc-OPE3), for which the thermal conductivity was reported by Mosso et al.[7] in agreement with observations by Cui et al.[8] . The protocol to measure Seebeck coefficient was applied also to this molecule yielding 7.9 ± 4 μV/K. This is in agreement with literature values obtained for single molecules and self-assembled monolayers, namely 8.0 ± 0.8 μV/K[17] , 7.78 ± 0.34 μV/K [18], and 9.7 ± 0.2 μV/K [19].

This makes SAc-OPE3 the second molecule for which the entire zT was measured using this protocol, yielding zT ≃ $2x10^{-5}$.

Bibliography

1. Moreno-García, P. *et al.* Single-molecule conductance of functionalized oligoynes: length dependence and junction evolution. *J Am Chem Soc* **135**, 12228–12240 (2013).

2. Huang, B. *et al.* Controlling and Observing Sharp-Valleyed Quantum Interference Effect in Single Molecular Junctions. *J Am Chem Soc* **140**, 17685–17690 (2018).

3. Ozawa, H. *et al.* Synthesis and Single-Molecule Conductance Study of Redox-Active Ruthenium Complexes with Pyridyl and Dihydrobenzo [b] thiophene Anchoring Groups. *Chemistry–A European Journal* **22**, 12732–12740 (2016).

4. Leary, E. *et al.* The Role of Oligomeric Gold-Thiolate Units in Single-Molecule Junctions of Thiol-Anchored Molecules. *Journal of Physical Chemistry C* **122**, 3211–3218 (2018).

5. Dekkiche, H. *et al.* Electronic conductance and thermopower of single-molecule junctions of oligo(phenyleneethynylene) derivatives. *Nanoscale* **12**, 18908–18917 (2020).

6. O'Driscoll, L. J. & Bryce, M. R. A review of oligo(arylene ethynylene) derivatives in molecular junctions. *Nanoscale* **13**, 10668–10711 (2021).

7. Mosso, N. *et al.* Thermal Transport through Single-Molecule Junctions. *Nano Lett* **19**, 7614–7622 (2019).

8. Cui, L. *et al.* Thermal conductance of single-molecule junctions. *Nature* (2019) doi:10.1038/s41586-019-1420-z.

9. Cui, L. *et al.* Study of radiative heat transfer in Ångström- and nanometre-sized gaps. *Nat Commun* **8**, 14479 (2017).

10. Kloppstech, K. *et al.* Giant heat transfer in the crossover regime between conduction and radiation. *Nat Commun* **8**, 14475 (2017).

11. Segal, D., Nitzan, A. & Hänggi, P. Thermal conductance through molecular wires. *Journal of Chemical Physics* **119**, 6840–6855 (2003).

12. Losego, M. D., Grady, M. E., Sottos, N. R., Cahill, D. G. & Braun, P. V. Effects of chemical bonding on heat transport across interfaces. *Nat Mater* **11**, 502–506 (2012).

13. Meier, T. *et al.* Length-dependent thermal transport along molecular chains. *Phys Rev Lett* **113**, 1–5 (2014).

14. Tabatabaeikahangi, F. A theoretical study of thermoelectric efficiency and cooling power in organic molecular junctions. (PhD. Diss., Université de Lyon, 2021).

15. Han, H., Mérabia, S. & Müller-Plathe, F. Thermal transport at solid–liquid interfaces: High pressure facilitates heat flow through nonlocal liquid structuring. *J Phys Chem Lett* **8**, 1946–1951 (2017).

16. Sadeghi, H., Sangtarash, S. & Lambert, C. J. Oligoyne Molecular Junctions for Efficient Room Temperature Thermoelectric Power Generation. *Nano Lett* **15**, 7467–7472 (2015).

17. Miao, R. *et al.* Influence of Quantum Interference on the Thermoelectric Properties of Molecular Junctions. *Nano Lett* **18**, 5666–5672 (2018).

18. Chen, H. *et al.* Quantum interference enhanced thermopower in single-molecule thiophene junctions. *Chinese Chemical Letters* **33**, 523–526 (2022).

19. Charalambos, E., Thermopower and conductance of single-molecule junctions and atomic contacts. PhD Thesis, Universidad Autónoma de Madrid (2014). http://hdl.handle.net/10486/663944
